# Comparative bleeding risk between angiotensin II receptor blockers with high versus low P-glycoprotein inhibitory activity in patients with atrial fibrillation receiving direct oral anticoagulants

**DOI:** 10.1038/s41440-026-02712-7

**Published:** 2026-06-17

**Authors:** Seonji Kim, Seng Chan You, Seung Il Kim, Ji-Hwan Bae, Jong-Il Choi, Sungha Park

**Affiliations:** 1 Department of Biomedical Systems Informatics, Yonsei University College of Medicine, Seoul, South Korea; 2 Yonsei Institute for Digital Health, Yonsei University, Seoul, South Korea; 3 Medical & Pharmacovigilance Department, Daiichi Sankyo Korea Co. Ltd., Seoul, South Korea; 4 Division of Cardiology, Department of Internal Medicine, Korea University College of Medicine and Korea University Anam Hospital, Seoul, South Korea; 5 Division of Cardiology, Severance Cardiovascular Hospital, Yonsei University College of Medicine, Seoul, South Korea

**Keywords:** Direct oral anticoagulants, Hypertension, P-glycoprotein inhibitor, Gastrointestinal bleeding, Intracranial haemorrhage

## Abstract

Direct oral anticoagulants (DOACs) are frequently co-prescribed with angiotensin II receptor blockers (ARBs) in atrial fibrillation (AF). Because ARBs differ in P-glycoprotein (P-gp) inhibition, bleeding risk may vary. We compared major bleeding when DOACs were combined with telmisartan (high P-gp inhibition) versus olmesartan (low P-gp inhibition). We conducted a nationwide cohort study from 2016–2023 in Korea. We identified AF patients who started a DOAC with telmisartan or olmesartan. The primary outcome was a composite of intracranial hemorrhage and gastrointestinal bleeding. Secondary outcomes included each component of the primary outcome and ischemic stroke. Hazard ratios (HRs) and 95% confidence intervals (CIs) were estimated using Cox proportional hazards models. Among DOAC-treated patients with AF, 27,851 and 20,010 patients were included in the telmisartan and olmesartan groups, respectively. The incidence rates of the primary outcome were 9.13 per 1000 person-years in the telmisartan group and 6.69 in the olmesartan group (HR 1.35, 95% CI 1.11–1.65). Concomitant use of telmisartan was associated with a higher risk of gastrointestinal bleeding (1.49, 1.17–1.89), but not with intracranial hemorrhage (1.06, 0.74–1.54) or ischemic stroke (0.94, 0.78–1.14). Among patients with AF using DOACs, concomitant use of ARBs with high P-gp inhibitory activity was associated with a significantly higher risk of major bleeding and gastrointestinal bleeding compared–ARBs with low P-gp inhibitory activity. These findings suggest that differences in P-gp inhibitory activity among ARBs may influence bleeding risk in clinical practice when co-administered with DOACs.

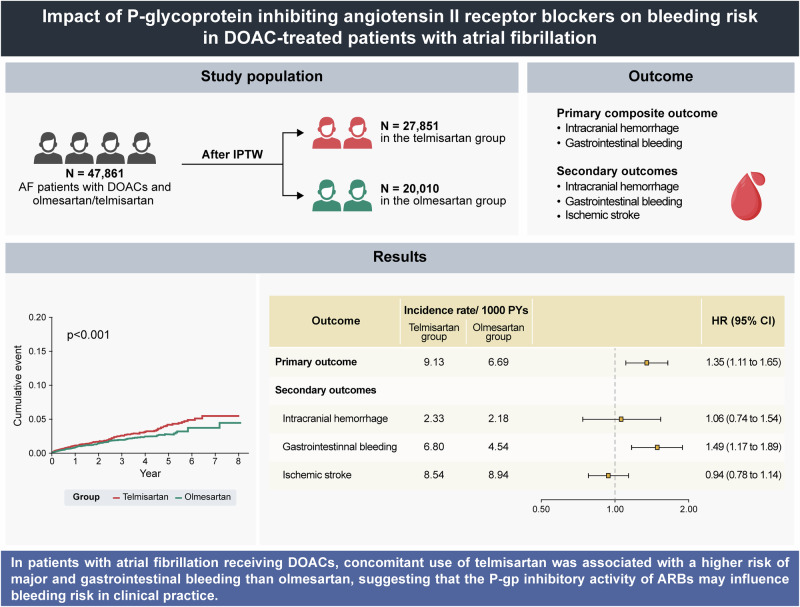

## Introduction

Atrial fibrillation (AF) is the most common type of arrhythmia, with hypertension being the most prevalent comorbidity affecting ~70–89% of AF patients worldwide [1–4]. Hypertension is a major risk factor for both thromboembolic and bleeding events, making optimal blood pressure control crucial in AF management [5, 6]. Angiotensin II receptor blockers (ARBs) are widely used antihypertensive agents because of their durability, blood pressure lowering efficacy, and favorable side effect profile [7–9]. Direct oral anticoagulants (DOACs) are preferred for stroke prevention in AF due to their convenience, fixed dosing, and lower bleeding risk compared to vitamin K antagonists [10]. Despite the advantages of DOACs, patients with AF often have multiple comorbidities and are prescribed several medications, placing them at a potentially increased risk of bleeding [11, 12].

Due to their shared excretion via P-glycoprotein (P-gp), all DOACs may exhibit clinically significant interactions with P-gp inhibitors, potentially affecting their anticoagulant activity [12]. The European Heart Rhythm Association (EHRA) published a practical guide on the use of DOACs in patients with AF, outlining specific drug–drug interactions involving P-gp inhibitors and providing recommendations for their management [10]. Since the publication of the EHRA guideline, accumulating evidence has confirmed that certain P-gp inhibitors are high-risk medications when co-administered with DOACs, leading to a significantly increased bleeding risk [13, 14]. Notably, within the ARB class, there is considerable variability in P-gp inhibitory properties. ARBs with high P-gp inhibitory activity may reduce the clearance of DOACs, thereby elevating the risk of bleeding compared to ARBs with low or minimal P-gp inhibitory effects [15–17]. Prior studies have demonstrated that telmisartan inhibits intestinal P-gp–mediated transport of digoxin, whereas olmesartan shows minimal interaction with P-gp and has been reported to neither inhibit digoxin transport nor activate ATPase activity in hMDR1-expressing membranes [16–18]. Despite the widespread use of ARBs in patients with AF, limited evidence exists regarding the differential impact of P-gp inhibitory activity among antihypertensive agents on DOAC-related bleeding risk.

Therefore, we compared the risk of composite bleeding outcomes between patients treated with DOACs combined with telmisartan, which has high P-gp inhibitory activity among ARBs, and olmesartan, which has low P-gp inhibitory activity among ARBs.

Point of view

- **Clinical relevance**: Among patients with AF receiving DOACs, concomitant use of telmisartan, which has high P-glycoprotein inhibitory activity, was associated with a higher risk of major bleeding and gastrointestinal bleeding than olmesartan, which has low P-glycoprotein inhibitory activity.
- **Future direction**: Prospective and mechanistic studies including P-glycoprotein genetic polymorphisms are needed to confirm causality and to clarify the bleeding risk associated with DOAC and ARBs.
- **Consideration for the Asian population**: Because Asian patients with AF carry a higher baseline risk of anticoagulation-related bleeding and ARBs are widely co-prescribed with DOACs, selecting an antihypertensive agent with low drug–interaction potential may be especially relevant in clinical practice.

## Methods

### Data sources

We retrieved the study data from the Korea Health Insurance Review and Assessment Service (HIRA) database. The Korean government operates a mandatory single-payer health insurance system. The HIRA database is a repository of claims data covering over 50 million people and ~99% of the Korean population [19]. This database includes comprehensive information on patient demographics, diagnoses, and drug prescriptions. Diagnostic information is coded according to the International Classification of Diseases, 10th Revision (ICD-10). This study adhered to the Strengthening the Reporting of Observational Studies in Epidemiology (STROBE) guidelines.

### Ethics approval

This study was approved by the Institutional Review Board of the Severance Hospital (IRB No. 4-2024-0016). The requirement for informed consent was waived because of the retrospective nature of the study. The study protocol in this study was registered at https://cris.nih.go.kr (registration number, KCT 0010330).

### Study design and study population

We conducted a nationwide, population-based, retrospective cohort study using Korean nationwide claims data from January 2016 to September 2023. We enrolled patients aged ≥19 years with AF who were received concomitant use of olmesartan or telmisartan at the time of DOAC initiation. Individuals with AF were identified based on at least two outpatient or one inpatient visits. The accuracy of AF identification has been assessed in earlier research, with a reported positive predictive value (PPV) of 94.1% [20, 21]. The study population included only those with an observation period of at least one year prior to the index date. Patients were excluded if patients 1) were treated with previous oral anticoagulants; 2) had a diagnosis of end-stage renal disease; 3) had a history of pulmonary embolism, deep vein thrombosis, or hip replacement surgery; 4) had experienced intracranial hemorrhage, gastrointestinal bleeding, or ischemic stroke prior to the index date; or 5) had received ARBs within 60 days before the index date. A flow chart is shown in Supplementary Fig. 1.

DOACs included apixaban, dabigatran, edoxaban, and rivaroxaban. The target group comprised patients receiving concomitant use of telmisartan and DOACs. Olmesartan was defined as the active comparator because it shares similar efficacy in BP lowering with telmisartan and minimal act as a P-gp inhibitor.

### Outcome definitions and covariates

The primary outcome was a composite of intracranial hemorrhage and gastrointestinal bleeding. The secondary outcomes included individual outcomes of the primary outcome, and ischemic stroke. The diagnostic accuracy of intracranial hemorrhage (PPV: 81.4%), gastrointestinal bleeding (PPV: 92.0%), and ischemic stroke (PPV: 92.2%) has been previously validated in studies utilizing data from the claims data in Korea [4, 21, 22]. The study population was censored at the earliest occurrence of death, the last date of the observation period, drug discontinuation, use of comparator drugs in the target group, or use of target drugs in the comparator group.

Covariates were used to balance the target and comparator groups and included patient demographics (age, and sex), index year, exposure drugs (drug type. dosage of DOACs, and dosage of olmesartan or telmisartan), prior comorbidities and co-medications within 3 months or 365 days before the index date, and the scores (CHA_2_DS_2_-VASc score, Charlson Comorbidity Index and, HAS-BLED score) over 365 days before the index date. Drug dosage was calculated using the Anatomical Therapeutic Chemical/Defined Daily Dose (ATC/DDD) methodology developed by the World Health Organization (WHO). The DDD is defined as the assumed average maintenance dose per day for a drug used for its main indication in adults [23]. Drug dosage was calculated by dividing the average prescribed daily dose by the WHO-assigned DDD for each drug. Details on the definitions of clinical outcomes, comorbidities, and clinical risk scores are outlined in Supplementary Table 1.

### Statistical analysis

A descriptive analysis was conducted to present the baseline characteristics. Means and standard deviations were used to describe continuous variables, and frequencies with percentages were used for categorical variables. To minimize potential bias arising from imbalances in observed covariate distribution between each treatment group, we applied inverse probability of treatment weighting (IPTW). To mitigate the influence of extreme weights, we applied weight stabilization and truncation at the 1st and 99th percentiles after evaluating their distribution across treatment groups. For reporting purposes, the weighted sample sizes were rounded to the nearest integer. Standardized mean differences (SMDs) was used to compare characteristics before and after propensity score (PS) method, and a SMD of less than 0.1 was considered acceptable.

Sensitivity analyses were performed to evaluate the robustness of the findings across variations in the time-at-risk definitions and PS methodologies. Four time-at-risk windows were considered. The first was during treatment, where patients were followed until treatment discontinuation. The remaining three used an intention-to-treat (ITT) approach, with follow-up periods of 1 year, 1.5 years, and 2 years from treatment initiation. Second, we applied four different PS-based methods: IPTW, 1:1 PS matching, PS stratification (10 strata), and inverse probability of censoring weighting (IPCW). Furthermore, we additionally evaluated the risks of adverse events associated with antihypertensive drugs (angioedema, hyperkalemia, hypotension, and syncope) and negative control outcomes (non-infectious pneumonitis, cancer, and urinary tract infections).

We conducted subgroup analyses by stratifying the study population according to the following factors: age (<65 years, 65–74 years, and ≥75 years), sex, CHA_2_DS_2_-VASc score, HAS-BLED score, history of heart failure, history of renal disease (excluding end-stage renal disease), comedications (use of P2Y₁₂ inhibitors, use of other antiplatelet agents, and the number of antihypertensive drug classes), medication adherence (≥0.8 vs <0.8), and type of DOAC. Medication adherence was calculated as the proportion of days covered, defined by dividing the number of days the medication was supplied by the total number of days in the observation period [24]. In addition, we performed interaction analyses by including interaction terms in the models to assess potential effect modification. Survival analysis was performed using the Kaplan–Meier analysis and the log-rank test. We estimated the incidence rates per 1000 person-years, hazard ratios (HR) and 95% confidence intervals (CIs) using the Cox proportional hazards regression model. The threshold for statistical significance level was defined as a two-sided *p*-value of 0.05. Database construction and statistical analysis were performed using SAS Enterprise Guide version 7.1 (SAS Institute, Cary, NC, USA) and R software version 3.5.1 (R Foundation for Statistical Computing).

## Results

### Baseline characteristics

Among 345,181 non-valvular AF patients initiating DOAC therapy, 47,861 (13.87%) who were newly prescribed either telmisartan (*n* = 27,946) or olmesartan (*n* = 19,915) without prior use of other ARBs within 60 days before the index date were included in the study. Before IPTW, the dosage of olmesartan or telmisartan, measured in DDD, was higher in the telmisartan group (1.06) than in the olmesartan group (0.96; SMD = 0.33). After IPTW, 27,851 and 20,010 patients were selected, and the SMDs for all baseline characteristics between telmisartan and olmesartan groups were <0.1, indicating balance in baseline characteristics between groups (Table 1, Supplementary Fig. 2). The most frequently use in DOACs were edoxaban (36.34% and 36.55%) and apixaban (29.38% and 29.48%) in the telmisartan and olmesartan groups. The CHA₂DS₂-VASc score, Charlson Comorbidity Index, and HAS-BLED score were 3.73, 2.79, and 2.60, respectively, in both groups. The mean number of antihypertensive drug classes previously used was 1.38 and 1.39 in telmisartan and olmesartan groups.

**Table 1:** Baseline characteristics of patients before and after inverse probability of treatment weighting

	Before IPTW			After IPTW		
	Telmisartan group (*N* = 27,946)	Olmesartan group (*N* = 19,915)	SMD	Telmisartan group (*N* = 27,851)	Olmesartan group (*N* = 20,010)	SMD
Age, years	71.98 ± 10.41	72.05 ± 10.46	0.01	72.04 ± 10.45	72.05 ± 10.47	0.00
Female	11,729 (42.00)	8667 (43.50)	0.03	11,890 (42.69)	8568 (42.80)	0.00
DOAC use						
Dabigatran	1595 (5.70)	1024 (5.10)	0.12	1522 (5.46)	1086 (5.43)	0.01
Rivaroxaban	8461 (30.30)	5333 (26.80)	0.08	8026 (28.82)	5713 (28.55)	0.01
Apixaban	8268 (29.60)	5731 (28.80)	0.02	8182 (29.38)	5898 (29.48)	0.00
Edoxaban	9622 (34.40)	7827 (39.30)	0.10	10,121 (36.34)	7313 (36.55)	0.00
Dosage of DOACs^a^	0.76 ± 0.26	0.75 ± 0.26	0.04	0.75 ± 0.26	0.75 ± 0.26	0.01
Dosage of olmesartan or telmisartan^a^	1.06 ± 0.32	0.96 ± 0.30	0.33	1.03 ± 0.29	1.03 ± 0.36	0.02
Comorbidities						
Asthma	5557 (19.90)	4057 (20.40)	0.01	5616 (20.16)	4057 (20.28)	0.00
Cardiovascular disease	19,546 (69.90)	14,024 (70.40)	0.01	19,568 (70.26)	14,089 (70.41)	0.00
Cancer	1992 (7.10)	1361 (6.80)	0.01	1952 (7.01)	1404 (7.02)	0.00
Chronic obstructive pulmonary disease	1385 (5.00)	909 (4.60)	0.02	1346 (4.83)	976 (4.88)	0.00
Dementia	610 (2.20)	422 (2.10)	0.00	604 (2.17)	433 (2.16)	0.00
Diabetes mellitus	9318 (33.30)	6833 (34.30)	0.02	9419 (33.82)	6792 (33.94)	0.00
Dyslipidemia	22,904 (82.00)	16,364 (82.20)	0.01	22,851 (82.05)	16,410 (82.01)	0.00
Gastric ulcer	5542 (19.80)	3996 (20.10)	0.01	5564 (19.98)	3987 (19.93)	0.00
Hypertension	27,738 (99.30)	19,799 (99.40)	0.02	27,662 (99.32)	19,877 (99.34)	0.00
Days with Hypertension within 1-year before index date	295.68 ± 96.47	294.00 ± 96.73	0.02	295.02 ± 97.08	294.98 ± 95.72	0.00
Hypotension	444 (1.60)	291 (1.50)	0.01	426 (1.53)	303 (1.52)	0.00
Liver disease	11,902 (42.60)	7999 (40.20)	0.05	11,611 (41.69)	8328 (41.62)	0.00
Parkinson’s disease	401 (1.40)	282 (1.40)	0.02	402 (1.44)	290 (1.45)	0.00
Renal disease other than end stage renal disease	3560 (12.70)	2693 (13.50)	0.02	3669 (13.17)	2653 (13.26)	0.00
Clinical risk scores						
CHA_2_DS_2_-VASc score	3.71 ± 1.46	3.74 ± 1.46	0.02	3.73 ± 1.46	3.73 ± 1.46	0.00
Charlson Comorbidity Index	2.79 ± 2.17	2.76 ± 2.15	0.02	2.79 ± 2.17	2.79 ± 2.16	0.00
HAS-BLED score	2.61 ± 1.02	2.58 ± 1.03	0.02	2.60 ± 1.03	2.60 ± 1.03	0.00
Concomitant drugs						
Antiarrhythmic drugs	10,807 (38.70)	7702 (38.70)	0.00	10,799 (38.77)	7766 (38.81)	0.00
Antihypertensive drugs^a^						
ARB/ACEi other than olmesartan or telmisartan	1.07 ± 0.66	1.03 ± 0.64	0.06	1.07 ± 0.67	1.03 ± 0.62	0.06
Beta blockers	0.36 ± 0.26	0.37 ± 0.28	0.04	0.37 ± 0.27	0.37 ± 0.27	0.00
Calcium channel blockers	0.96 ± 0.45	1.01 ± 0.45	0.11	0.97 ± 0.47	1.00 ± 0.46	0.06
Diuretics	0.69 ± 0.65	0.77 ± 0.83	0.11	0.74 ± 0.87	0.73 ± 0.73	0.01
Number of classes for previous use of antihypertensive drugs	1.35 ± 0.65	1.44 ± 0.73	0.02	1.38 ± 0.67	1.39 ± 0.70	0.00
Corticosteroid in past 90 days^a^	3.31 ± 19.50	3.12 ± 7.05	0.01	3.26 ± 17.00	3.18 ± 7.71	0.01
P-gp/CYP3A4 interacting drugs	16,719 (59.80)	11,776 (59.10)	0.01	16,598 (59.60)	11,935 (59.65)	0.00
Lipid lowering therapies	18,695 (66.90)	13,419 (67.40)	0.01	18,704 (67.16)	13,448 (67.21)	0.00
Nonsteroidal anti-inflammatory drugs	22,554 (80.70)	15,923 (80.00)	0.02	22,397 (80.42)	16,090 (80.41)	0.00
P2Y12 inhibitors and other antiplatelet drugs	17,704 (63.40)	13,146 (66.00)	0.06	17,979 (64.45)	12,929 (64.61)	0.00
Proton-pump inhibitors	14,538 (52.00)	10,430 (52.40)	0.01	14,554 (52.25)	10,481 (52.38)	0.00
Insulin	2794 (10.00)	2224 (11.20)	0.04	2964 (10.64)	2173 (10.86)	0.01
Oral hypoglycemic agents	9224 (33.00)	6732 (33.80)	0.02	9305 (33.41)	6701 (33.49)	0.00
SSRI/SNRI	2139 (7.70)	1567 (7.90)	0.01	2163 (7.76)	1561 (7.80)	0.00
Vitamin K antagonists	1 (0.00)	1 (0.00)	0.00	1 (0.00)	1 (0.00)	0.00
Amiodarone	3402 (12.17)	2515 (12.63)	0.01	3408 (12.24)	2569 (12.84)	0.02
Diltiazem	3717 (13.30)	2790 (14.01)	0.02	3695 (13.27)	2876 (14.37)	0.03
Verapamil	1104 (3.95)	751 (3.77)	0.01	1082 (3.89)	767 (3.84)	0.00

Values for categorical variables are presented as n (%), and continuous variables are presented as mean ± standard deviation

*IPTW* inverse probability of treatment weighting, *SMD* standardized mean difference, *DOAC* direct oral anticoagulants, *ARB* angiotensin receptor blocker, *ACEi* angiotensin converting enzyme inhibitor, *P-gp* p-glycoprotein, *CYP* cytochrome P 450, *SSRI* selective serotonin reuptake inhibitors, *SRNI* serotonin and norepinephrine reuptake inhibitors

^a^Assessed by defined daily dose

### Association of concomitant use of DOACs and olmesartan

**Fig. 1:**
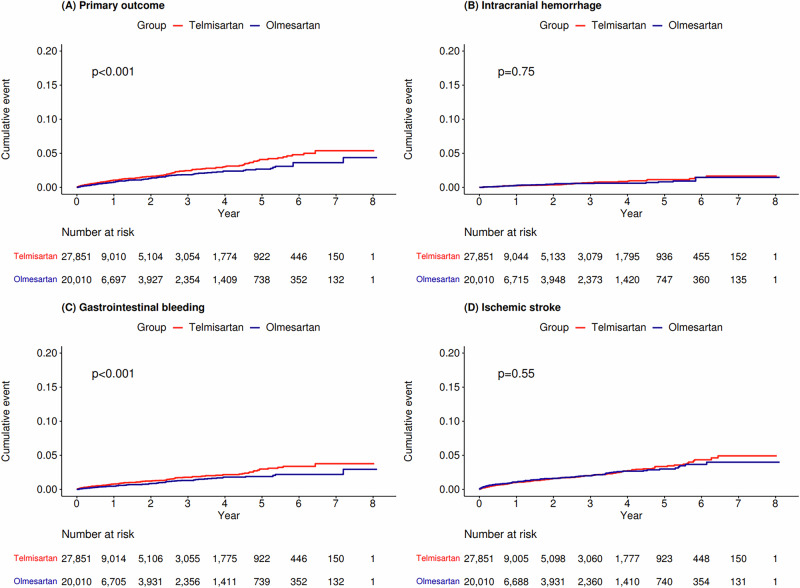
Kaplan–Meier curves for primary and secondary outcomes in telmisartan and olmesartan groups

After ITPW, the incidence rates of primary outcome were in 9.13 per 1000 person-years taking DOACs plus telmisartan and 6.69 per 1000 person-years taking DOACs plus olmesartan, respectively (HR 1.35 [95% CI 1.11–1.65]; Table 2). The concomitant use of telmisartan was associated with significantly higher gastrointestinal bleeding (1.49 [1.17–1.89]), but not with intracranial hemorrhage (1.06 [0.74–1.54]), and ischemic stroke (0.94 [0.78–1.14]) in patients treated DOACs. The cumulative incidence curves for the 4 clinical outcomes are shown in Fig. 1.

**Table 2:** Hazard ratios for primary and secondary outcomes

	Telmisartan group			Olmesartan group			Hazard ratio (95% CI)
	PYs	No. of events	IR/ 1000 PYs	PYs	No. of events	IR/ 1000 PYs	
Composite of intracranial hemorrhage or gastrointestinal bleeding	29,430.06	269	9.13	22,017.98	147	6.69	1.35 (1.11–1.65)
Secondary outcomes							
Intracranial hemorrhage	29,573.61	69	2.33	22,117.56	48	2.18	1.06 (0.74–1.54)
Gastrointestinal bleeding	29,441.39	200	6.80	22,032.57	100	4.54	1.49 (1.17–1.89)
Ischemic stroke	29,430.96	251	8.54	22,013.06	197	8.94	0.94 (0.78–1.14)

*PYs* person years, *IRs* incidence rates, *CI* confidence interval

### Sensitivity analysis

**Fig. 2:**
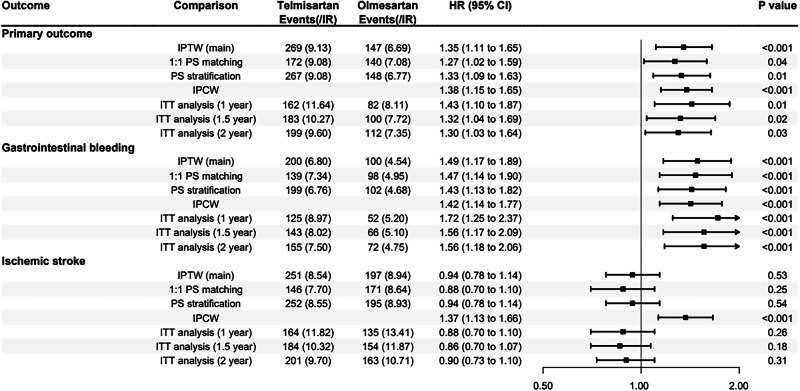
Risk of primary and secondary outcomes between telmisartan and olmesartan groups by times of risk definition and propensity score methods. IR incidence rate, HR hazard ratio, CI confidence interval, IPTW inverse probability of treatment weighting, PS propensity score, IPCW inverse probability of censoring weighting, ITT intention-to-treat

The DOACs dose and all covariate variables were balanced after PS matching and stratification (Supplementary Table 2). Across multiple time-at-risk definitions and PS approaches (Fig. 2), DOACs + telmisartan consistently increased the risk of major bleeding in the 1:1 PS matching (HR 1.27 [95% CI 1.02–1.59]), PS stratification (1.33 [1.09–1.63]), IPCW (1.38 [1.15–1.65]), ITT analysis until 1-year (1.43 [1.10–1.87]), ITT analysis until 1.5-year (1.32 [1.04–1.69]), and ITT analysis until 2-year (1.30 [1.03–1.64]). No significant differences in the risks of ischemic stroke were observed across all time window settings except IPCW.

In cases of antihypertensive drug-related adverse events, there were no significantly higher risks of angioedema (HR 1.17 [95% CI 0.59–2.32]), hyperkalemia (0.99 [0.88–1.10]), hypotension (0.93 [0.79–1.09]), or syncope (1.01 [0.90–1.14]) with the concomitant use of DOACs and telmisartan compared to DOACs and olmesartan (Supplementary Table 3). In the analysis of negative controls, there was no association with non-infectious pneumonitis (0.96 [0.74–1.24]), cancer (1.05 [0.97–1.12]), or urinary tract infections (0.98 [0.91–1.06]; Supplementary Table 4).

### Subgroup analysis

**Fig. 3:**
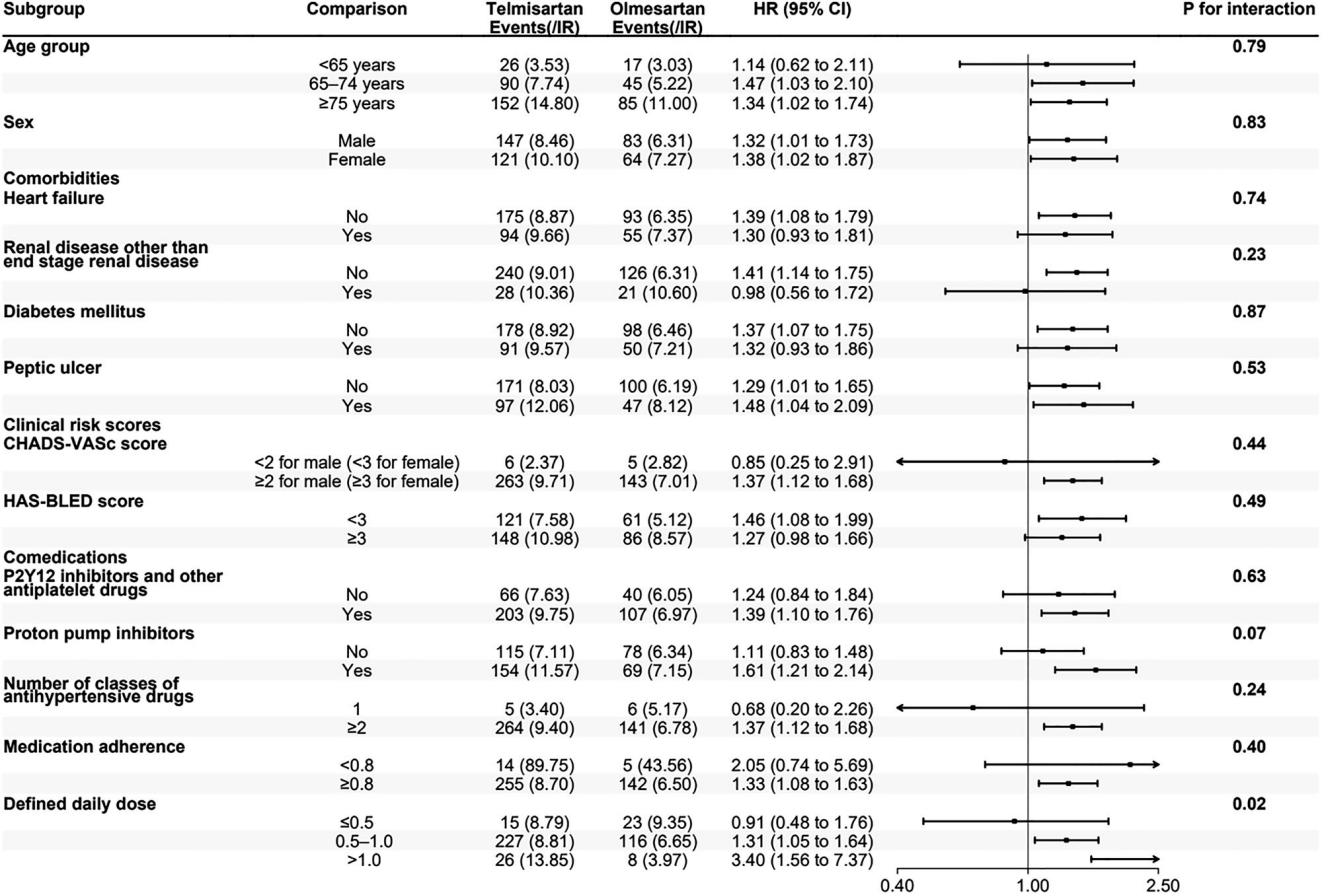
Subgroup analyses of major bleeding risk in telmisartan versus olmesartan groups. IR incidence rate, HR hazard ratio

Subgroup analyses for major bleeding are shown in Fig. 3. No significant interactions were observed across subgroups (all *P* for interaction >0.05). In subgroups with patients with history of renal disease, CHA₂DS₂-VASc score <2 for men (<3 for women), those receiving a single class of antihypertensive drugs, and presence of renal disease, the HRs were below 1; however, the 95% confidence intervals were wide and not statistically significant. Supplementary Table 5 presents the results for ischemic stroke risk, showing no statistically significant findings across all subgroups. Supplementary Table 6 presents subgroup analyses according to individual DOAC agents. Overall, the risk of major bleeding was generally consistent across DOACs, although most comparisons did not reach statistical significance and no significant interactions were observed across subgroups (*P* for interaction >0.05).

## Discussion

In this large-scale retrospective cohort study, we evaluated drug-drug interactions between DOACs and telmisartan in patients with atrial fibrillation, assessing the risk of intracranial hemorrhage and gastrointestinal bleeding using Korean nationwide claims data. The concomitant use of telmisartan was associated with a significantly higher risk of major bleeding (HR 1.35 [95% CI 1.11–1.65]) and gastrointestinal bleeding (1.49 [1.17–1.89]) compared to patients receiving olmesartan. However, the risks of intracranial hemorrhage (1.06 [0.74–1.54]), and ischemic stroke (0.94 [0.78–1.14]) were not significantly different. Sensitivity analyses and the negative control outcome yielded consistent results, supporting the robustness of the observed associations.

Given their widespread use in AF, a clear understanding of potential drug–drug interactions with DOACs, particularly those involving P-gp inhibitors, remains essential [13, 25]. All currently available DOACs are substrates of P-gp transporters, which are primarily expressed in the gastrointestinal tract and, to a lesser extent, in the liver and renal tubules. These transporters contribute to both intestinal excretion and renal clearance of DOACs [13, 25, 26]. Concomitant use of P-gp inhibitors can elevate plasma levels of DOACs, thereby increasing bleeding risk. Several medications commonly prescribed in patients with AF, including some antihypertensive agents, have P-gp inhibitory properties and may alter DOAC pharmacokinetics. Telmisartan has been reported in pharmacology studies to inhibit P-gp transport, whereas olmesartan shows minimal P-gp inhibition [15–17]. By elevating DOAC plasma levels, telmisartan may predispose to bleeding events.

Our findings extend prior evidence on pharmacokinetic interactions between DOACs and concomitant medications. Several observational studies and meta-analyses shown that P-gp inhibitors are linked to increased bleeding risk in DOAC-treated AF patients [13, 14, 26–28]. Wong et al. reported elevated gastrointestinal bleeding risks with digoxin (HR 1.30 [95% CI 1.13–1.51]), diltiazem (1.18 [1.01–1.39]), clarithromycin (4.98 [3.87–6.42]), and fluconazole (2.38 [1.23–4.59]) in a Hong Kong cohort of 22,568 DOAC users [14]. In a nationwide Belgian cohort study, concomitant use of P-gp/CYP3A4 inhibitors was associated with increased risks of major bleeding among DOAC users, including amiodarone (1.27 [1.21–1.34]), diltiazem (1.28 [1.13–1.46]), ticagrelor (1.50 [1.20–1.87]), verapamil (1.36 [1.03–1.80]), and clarithromycin (1.55 [1.14–2.11]) [13]. Meta-analysis of randomized controlled trials and observational cohort studies also found that concomitant P-gp/CYP3A4 inhibitors collectively conferred approximately a 10% higher risk of major bleeding (95% CI 1.01–1.19) [26]. However, most of prior studies frequently grouped diverse P-gp inhibitors together, but when examined at the individual drug level, sample sizes were insufficient limiting statistical power [13, 26–28]. Furthermore, the lack of active comparator designs and the heterogeneity among drug classes further hindered the assessment of clinical outcomes [26]. To assess the clinical relevance of these pharmacokinetic interactions, we employed an active comparator design using olmesartan, which is one of the ARBs with minimal P-gp inhibitory activity. Our findings showed that patients initiating DOACs in combination with telmisartan had significantly higher risks of major bleeding and gastrointestinal bleeding compared to those taking olmesartan. These results suggest that differences in P-gp inhibitory activity among ARBs may have clinically meaningful implications for bleeding risk in patients receiving DOAC therapy. In contrast, intracranial hemorrhage event rates were relatively low, which may have limited statistical power to detect risk differences. Within the ARB class, certain agents such as candesartan cilexetil and irbesartan have been shown to inhibit P-gp-mediated transport in experimental studies [16, 29]. The magnitude of inhibition varies substantially across agents, with telmisartan demonstrating the strongest inhibitory activity. However, as these findings are largely based on preclinical evidence and our analysis directly compared only two ARBs, further studies are warranted to confirm causality.

To our knowledge, this is the largest study to date to evaluate drug–drug interactions between DOACs and specific P-gp inhibiting agents representing routine clinical practice. Second, an active comparator design was employed to minimize confounding by indication and enable a clinically meaningful comparison between antihypertensive agents with similar therapeutic indications but differing P-gp inhibitory properties. Third, we performed comprehensive sensitivity analyses to test the robustness and specificity of our results, applying multiple time-at-risk windows, advanced PS-based methods including IPCW, and additional assessments of antihypertensive-related adverse events and negative control outcomes.

Despite its strengths, this observational cohort study has several limitations. First, misclassification of diagnoses and outcomes is a potential limitation of administrative data. However, disease definitions based on diagnostic, procedural, and prescription codes likely minimized this risk. Furthermore, previous validation studies have reported PPV over 80% for AF and clinical outcomes [4, 20–22]. Second, prescription records may not fully reflect actual medication use. Third, although we adjusted for approximately 40 confounding factors, residual confounding remains possible due to unmeasured clinical parameters such as laboratory results (e.g., renal function and hemoglobin levels), body weight, clinical severity and blood pressure. Longitudinal changes in renal function (e.g., creatinine clearance) and achieved blood pressure during follow-up could not be assessed using claims data. Given that renal function directly affects DOAC clearance, unmeasured or time-varying changes in renal function may have influenced drug exposure and bleeding risk. Therefore, this limitation should be considered when interpreting the findings. Fourth, while our findings demonstrate a significant difference in bleeding risk between the telmisartan and olmesartan groups, this variation may not be solely attributable to differences in P-gp inhibitory activity. It is possible that the inherent antihypertensive potency of each agent also played a role in the observed outcomes. Fifth, genetic polymorphisms affecting P-glycoprotein activity were not assessed because genetic information was unavailable in the claims database. Variability in transporter and metabolic activity related to these polymorphisms may influence DOAC pharmacokinetics and bleeding risk. Further studies incorporating genomic data are warranted to clarify the potential influence of P-gp polymorphisms on DOAC-related bleeding risk.

### Perspective of Asia

Hypertension is the most prevalent comorbidity in AF, and ARBs are widely used antihypertensive agents, making the co-administration of an ARB with a DOAC an extremely common scenario in routine practice. Asian patients with AF are known to carry a higher baseline risk of anticoagulation-related bleeding than non-Asian patients [30]. In this context, minimizing avoidable pharmacokinetic drug–drug interactions may be of particular clinical importance. Using Korean nationwide claims data, this study provides Asia-specific real-world evidence that concomitant use of telmisartan, which has high P-gp inhibitory activity, is associated with a higher risk of major bleeding than olmesartan, which has low P-gp inhibitory activity. These findings suggest that selecting an antihypertensive agent with low drug–interaction potential may be especially relevant in Asian clinical practice, where the burden of both AF and anticoagulation-related bleeding continues to grow.

## Conclusions

In this nationwide retrospective cohort study, we found that among patients with AF initiating DOAC therapy, concomitant use of telmisartan, which is one of the ARBs with stronger P-gp inhibitory activity, was associated with a significantly higher risk of major bleeding and gastrointestinal bleeding compared to olmesartan, which has minimal P-gp inhibitory properties. These real-world findings suggest that differences in P-gp mediated drug interactions among antihypertensive agents may be associated with variation in bleeding risk in DOAC-treated patients. Antihypertensive agents with lower interaction potential may be considered in patients receiving DOACs, particularly in those at increased risk of bleeding.

## Supplementary information

Supplementary Information

## Data Availability

This study used data from the Korea Health Insurance Review and Assessment Service (HIRA) (M20240509003). We acknowledge the Korea HIRA for granting access to the data used in this study.
