# Supplementary material for: Comparative bleeding risk between angiotensin II receptor blockers with high versus low P-glycoprotein inhibitory activity in patients with atrial fibrillation receiving direct oral anticoagulants
Source: Hypertens Res. 2026 Jun 17;49(8):2318–28. doi: 10.1038/s41440-026-02712-7 (PMC13437244; doi:10.1038/s41440-026-02712-7)
Supplement: Supplementary file 1 [file 41440_2026_2712_MOESM1_ESM.docx]

Supplementary Table 1. Definition of clinical outcomes, comorbidities, and clinical risk scores

|  | **Definitions** | **Codes or conditions** |
| --- | --- | --- |
| **Primary outcome** |  |  |
| Composite of intracranial hemorrhage  and gastrointestinal bleeding | Intracranial hemorrhage or gastrointestinal bleeding |  |
| **Secondary outcomes** |  |  |
| Ischemic stroke | Defined from admission diagnosis + brain imaging (CT or MRI) | Any primary admission diagnosis with I63 or I64  Brain imaging: HE101, HE201, HE501, HE135, HE235, HE535, HF101, HF201, HF102, HF202, HI101, HI201, HI501, HI135, HI235, HI535, HA451, HA461, HA471, HA531 |
| Intracranial bleeding | Defined from admission diagnosis + brain imaging (CT or MRI) | Any primary admission diagnosis with I60-I62  Brain imaging: HE101, HE201, HE501, HE135, HE235, HE535, HF101, HF201, HF102, HF202, HI101, HI201, HI501, HI135, HI235, HI535, HA451, HA461, HA471, HA531 |
| Gastrointestinal bleeding | Defined from admission diagnosis | Any primary admission diagnosis with K22.6, K25.0, K25.2, K25.4, K25.6, K26.0, K26.2, K26.4, K26.6, K27.0, K27.2, K27.4, K27.6, K28.0, K28.2, K28.4, K28.6, K29.0, K62.5, K92.0, K92.1, or K92.2 |
| **Comorbidities** |  |  |
| Asthma | Defined from diagnosis^*^ | ICD-10: J45, J46 |
| Myocardial infarction | Defined from diagnosis^*^ | ICD-10: I21, I22 |
| Transient ischemic attack | Defined from diagnosis^*^ | ICD-10: G45 |
| Heart failure | Defined from diagnosis^*^ | ICD-10: I50 |
| Ischemic heart disease  (without myocardial infarction) | Defined from diagnosis^*^ | ICD-10: I20, I23-I25 |
| Peripheral artery disease | Defined from diagnosis^†^ | ICD-10: I70, I73 |
| Cancer | Defined from diagnosis^*^ | ICD-10: C00-97 and V193 (rare intractable diseases [RIDs] diagnostic code for cancer) |
| Chronic obstructive pulmonary disease | Defined from diagnosis | Any admission diagnosis with J41-J44 |
| Dementia | Defined from diagnosis^*^ | ICD-10: F00-F03, G30.0, G30.1, G30.8, G30.9, G31.0, G31.82, A81.0 |
| Diabetes mellitus | Defined from diagnosis + treatment^†^ | ICD-10: E10, E11, E12, E13, E14  Treatment: all kinds of oral anti-diabetics (sulfonylureas, metformin, meglitinides, thiazolidinediones, dipeptidyl peptidase-4 inhibitors, and α-glucosidase inhibitors) and insulin |
| Dyslipidemia | Defined from diagnosis^*^ | ICD-10: E78 |
| Gastric ulcer | Defined from diagnosis^*^ | ICD-10: K25 |
| Hypertension | Defined from diagnosis + treatment^†^ | ICD-10: I10-I13, I15  Treatment: all kinds of antihypertensive drug (thiazide, loop diuretics, aldosterone antagonist, alpha-/beta-blocker, calcium-channel blocker, angiotensin-converting enzyme inhibitor, angiotensin II receptor blocker) |
| Hypotension | Defined from diagnosis^*^ | ICD-10: I95 (except I95.2) |
| Liver disease | Defined from diagnosis^*^ | ICD-10: K70, K72-K76, K71.3-K71.7 |
| Parkinson's disease | Defined from diagnosis^*^ | ICD-10: G20 |
| Renal disease | Defined from diagnosis^*^ | ICD-10: I13.1, N03, N05, N10-N19, Z49, Z94.0, Z99.2 |
| Venous thromboembolism | Defined from diagnosis | Any admission diagnosis with I80.2 |
| Deep vein thrombosis | Defined from diagnosis | Any admission diagnosis with I26 |
| **Clinical risk scores** |  |  |
| CHA_2_DS_2_-VASc score | Heart failure, hypertension, age ≥75 years, diabetes mellitus, previous stroke, systemic embolism or transient ischemic attack, vascular disease (prior myocardial infarction or peripheral artery disease) and female | |
| Charlson Comorbidity Index | Defined from diagnosis;  Weight 1: Chronic pulmonary disease (ICD-10: I27.8, I27.9, J40–J47, J60–J67, J68.4, J70.1, J70.3), rheumatologic disease (M05, M06, M31.5, M32, M34, M35.1, M35.3, M36.0), diabetes with chronic complications (E10.2–E10.5, E10.7, E11.2–E11.5, E11.7, E12.2–E12.5, E12.7, E13.2–E13.5, E13.7, E14.2–E14.5, E14.7), and renal disease (N18, N19, N25.0, Z49.0, Z49.2, Z94.0, Z99.2);  Weight 2: Congestive heart failure (I09.9, I11.0, I13.0, I13.2, I25.5, I42.0, I42.5–I42.9, I43, I50, P29.0), dementia (F00–F03, F05.1, G30, G31.1), mild liver disease (B18, K70.0–K70.3, K70.9, K71.3–K71.5, K71.7, K73, K74, K76.0, K76.2–K76.4, K76.8, K76.9, Z94.4), hemiplegia or paraplegia (G04.1, G11.4, G80.1, G80.2, G81, G82, G83.0–G83.4, G83.9), and any malignancy including lymphoma and leukemia, except malignant neoplasm of skin (C00–C26, C30–C34, C37–C41, C43, C45–C58, C60–C76, C81–C85, C88, C90–C97);  Weight 4: Moderate or severe liver disease (I85.0, I85.9, I86.4, I98.2, K70.4, K71.1, K72.1, K72.9, K76.5, K76.6, K76.7), AIDS/HIV (B20–B22, B24);  Weight 6: Metastatic solid tumor (C77–C80) | |
| HAS-BLED score | Hypertension, liver disease, renal disease, stroke history, bleeding history, age>65 years and drug use (concomitant use of non-steroidal anti-inflammatory drug or antiplatelet agent)^‡^ | |

^*^Comorbidities were established based on one inpatient or one outpatient records of ICD-10 codes in the database.

^†^Comorbidities were established based on one inpatient or two outpatient records of ICD-10 codes in the database.

^‡^As labile international normalized ratio and alcohol use were not available in claims database, they were not included in the scoring.

CT, computed tomography; MRI, magnetic resonance imaging; ICD-10, the International Classification of Diseases, 10th Revision.

Supplementary Table 2. Baseline characteristics of patients after propensity score matching and propensity score stratification

|  | Before propensity score methods | | | After propensity score matching | | | After propensity score stratification | | |
| --- | --- | --- | --- | --- | --- | --- | --- | --- | --- |
|  | Olmesartan group  (N=19,915) | Telmisartan group  (N=27,946) | SMD | Olmesartan group  (N=17,767) | Telmisartan group  (N=17,767) | SMD | Olmesartan group  (N=19,915) | Telmisartan group  (N=27,946) | SMD |
| Age, years, mean$\pm$SD | 72.05 ±10.46 | 71.98 ± 10.41 | 0.01 | 72.03 ±10.47 | 72.01 ± 10.48 | 0.00 | 72.06 ±10.45 | 72.08 ± 10.47 | 0.00 |
| Female | 8,667 (43.50) | 11,729 (42.00) | 0.03 | 7,627 (42.93) | 7,674 (43.19) | 0.01 | 8,506.97 (42.72) | 11,985.06 (42.89) | 0.00 |
| **DOAC use** |  |  |  |  |  |  |  |  |  |
| Dabigatran | 1,024 (5.10) | 1,595 (5.70) | 0.12 | 948 (5.34) | 928 (5.22) | 0.02 | 1,091.27 (5.48) | 1,503.36 (5.38) | 0.02 |
| Rivaroxaban | 5,333 (26.80) | 8,461 (30.30) | 0.08 | 5,016 (28.23) | 4,840 (27.24) | 0.02 | 5,736.80 (28.81) | 7,969.81 (28.52) | 0.01 |
| Apixaban | 5,731 (28.80) | 8,268 (29.60) | 0.02 | 5,185 (29.18) | 5,154 (29.01) | 0.00 | 5,864.11 (29.45) | 8,194.47 (29.32) | 0.00 |
| Edoxaban | 7,827 (39.30) | 9,622 (34.40) | 0.10 | 6,618 (37.25) | 6,845 (38.53) | 0.03 | 7,222.81 (36.27) | 10,278.35 (36.78) | 0.01 |
| Dosage of DOACs^*^ | 0.75 ±0.26 | 0.76 ± 0.26 | 0.04 | 0.75 ±0.26 | 0.75 ± 0.25 | 0.01 | 0.75 ±0.26 | 0.75 ± 0.26 | 0.01 |
| Dosage of Olmesartan or telmisartan^*^ | 0.96 ±0.30 | 1.06 ± 0.32 | 0.33 | 0.99 ±0.28 | 1.01 ± 0.26 | 0.06 | 1.01 ±0.31 | 1.03 ± 0.31 | 0.07 |
| **Comorbidities** |  |  |  |  |  |  |  |  |  |
| Asthma | 4,057 (20.40) | 5,557 (19.90) | 0.01 | 3,577 (20.13) | 3,596 (20.24) | 0.00 | 4,008.71 (20.13) | 5,673.13 (20.30) | 0.00 |
| Cardiovascular disease | 14,024 (70.40) | 19,546 (69.90) | 0.01 | 12,430 (69.96) | 12,532 (70.54) | 0.01 | 13,966.17 (70.13) | 19,709.77 (70.53) | 0.01 |
| Cancer | 1,361 (6.80) | 1,992 (7.10) | 0.01 | 1,232 (6.93) | 1,204 (6.78) | 0.01 | 1,394.71 (7.00) | 1,951.51 (6.98) | 0.00 |
| Chronic obstructive pulmonary disease | 909 (4.60) | 1,385 (5.00) | 0.02 | 835 (4.70) | 832 (4.68) | 0.00 | 953.29 (4.79) | 1,360.50 (4.87) | 0.00 |
| Dementia | 422 (2.10) | 610 (2.20) | 0.00 | 387 (2.18) | 384 (2.16) | 0.00 | 430.03 (2.16) | 616.90 (2.21) | 0.00 |
| Diabetes mellitus | 6,833 (34.30) | 9,318 (33.30) | 0.02 | 6,041 (34.00) | 6,101 (34.34) | 0.01 | 6,704.97 (33.67) | 9,526.05 (34.09) | 0.01 |
| Dyslipidemia | 16,364 (82.20) | 22,904 (82.00) | 0.01 | 14,556 (81.93) | 14,635 (82.37) | 0.01 | 16,320.07 (81.95) | 22,962.52 (82.17) | 0.01 |
| Gastric ulcer | 3,996 (20.10) | 5,542 (19.80) | 0.01 | 3,552 (19.99) | 3,590 (20.21) | 0.01 | 3,977.39 (19.97) | 5,603.86 (20.05) | 0.00 |
| Hypertension | 19,799 (99.40) | 27,738 (99.30) | 0.02 | 17,657 (99.38) | 17,659 (99.39) | 0.00 | 19,783.44 (99.34) | 27,755.88 (99.32) | 0.00 |
| Days with Hypertension within 1-year before index date | 294.00 ±96.73 | 295.68 ± 96.47 | 0.02 | 295.28 ±95.55 | 295.68 ± 96.47 | 0.01 | 295.49 ±95.40 | 294.50 ± 97.56 | 0.01 |
| Hypotension | 291 (1.50) | 444 (1.60) | 0.01 | 264 (1.49) | 253 (1.42) | 0.01 | 306.75 (1.54) | 424.33 (1.52) | 0.00 |
| Liver disease | 7,999 (40.20) | 11,902 (42.60) | 0.05 | 7,313 (41.16) | 7,278 (40.96) | 0.00 | 8,316.49 (41.76) | 11,605.39 (41.53) | 0.01 |
| Parkinson’s disease | 282 (1.40) | 401 (1.40) | 0.02 | 252 (1.42) | 271 (1.53) | 0.01 | 283.56 (1.42) | 405.53 (1.45) | 0.00 |
| Renal disease other than end stage renal disease | 2,693 (13.50) | 3,560 (12.70) | 0.02 | 2,325 (13.09) | 2,366 (13.32) | 0.01 | 2,601.84 (13.06) | 3,750.31 (13.42) | 0.01 |
| **Clinical risk scores** |  |  |  |  |  |  |  |  |  |
| CHA_2_DS_2_-VASc score | 3.74 ± 1.46 | 3.71 ± 1.46 | 0.02 | 3.73 ± 1.45 | 3.74 ± 1.46 | 0.01 | 3.73 ± 1.46 | 3.74 ± 1.47 | 0.01 |
| Charlson Comorbidity Index | 2.76 ± 2.15 | 2.79 ± 2.17 | 0.02 | 2.76 ± 2.15 | 2.77 ± 2.16 | 0.00 | 2.60 ± 1.03 | 2.61 ± 1.03 | 0.00 |
| HAS-BLED score | 2.58 ± 1.03 | 2.61 ± 1.02 | 0.02 | 2.59 ± 1.03 | 2.59 ± 1.03 | 0.00 | 2.78 ± 2.16 | 2.80 ± 2.17 | 0.01 |
| **Concomitant drugs** |  |  |  |  |  |  |  |  |  |
| Antiarrhythmic drugs | 7,702 (38.70) | 10,807 (38.70) | 0.00 | 6,853 (38.57) | 6,889 (38.77) | 0.00 | 7,710.09 (38.72) | 10,864.06 (38.88) | 0.00 |
| Antihypertensive drugs^*^ |  |  |  |  |  |  |  |  |  |
| ARB/ACEi other than olmesartan or telmisartan | 1.03 ± 0.64 | 1.07 ± 0.66 | 0.06 | 1.02 ± 0.60 | 1.06 ± 0.67 | 0.06 | 1.03 ± 0.62 | 1.07 ± 0.67 | 0.06 |
| Beta blockers | 0.37 ± 0.28 | 0.36 ± 0.26 | 0.04 | 0.37 ± 0.27 | 0.37 ± 0.28 | 0.00 | 0.37 ± 0.27 | 0.37 ± 0.27 | 0.00 |
| Calcium channel blockers | 1.01 ± 0.45 | 0.96 ± 0.45 | 0.11 | 1.00 ± 0.44 | 0.99 ± 0.46 | 0.02 | 1.00 ± 0.46 | 0.97 ± 0.47 | 0.06 |
| Diuretics | 0.77 ± 0.83 | 0.69 ± 0.65 | 0.11 | 0.73 ± 0.87 | 0.73 ± 0.57 | 0.00 | 0.73 ± 0.73 | 0.74 ± 0.87 | 0.01 |
| Number of classes for previous use of antihypertensive drugs | 1.44 ± 0.73 | 1.35 ± 0.65 | 0.02 | 1.40 ± 0.70 | 1.43 ± 0.69 | 0.05 | 1.37 ± 0.69 | 1.40 ± 0.68 | 0.04 |
| Corticosteroid in past 90 days^*^ | 3.12 ± 7.05 | 3.31 ± 19.50 | 0.01 | 3.12 ± 7.29 | 3.03 ± 4.44 | 0.01 | 3.18 ± 7.71 | 3.26 ± 17.00 | 0.01 |
| P-gp/CYP3A4 interacting drugs | 11,776 (59.10) | 16,719 (59.80) | 0.01 | 10,549 (59.37) | 10,573 (59.51) | 0.00 | 11,872.43 (59.62) | 16,648.10 (59.57) | 0.00 |
| Lipid lowering therapies | 13,419 (67.40) | 18,695 (66.90) | 0.01 | 11,915 (67.06) | 12,013 (67.61) | 0.01 | 13,349.01 (67.03) | 18,816.63 (67.33) | 0.00 |
| Nonsteroidal anti-inflammatory drugs | 15,923 (80.00) | 22,554 (80.70) | 0.02 | 14,245 (80.18) | 14,229 (80.09) | 0.00 | 16,031.90 (80.50) | 22,445.07 (80.32) | 0.01 |
| P2Y12 inhibitors and other antiplatelet drugs | 13,146 (66.00) | 17,704 (63.40) | 0.06 | 11,516 (64.82) | 11,686 (65.77) | 0.02 | 12,780.01 (64.17) | 18,104.53 (64.78) | 0.01 |
| Proton-pump inhibitors | 10,430 (52.40) | 14,538 (52.00) | 0.01 | 9,287 (52.27) | 9,314 (52.42) | 0.00 | 10,411.96 (52.28) | 14,626.94 (52.34) | 0.00 |
| Insulin | 2,224 (11.20) | 2,794 (10.00) | 0.04 | 1,894 (10.66) | 1,998 (11.25) | 0.02 | 2,106.34 (10.58) | 3,057.43 (10.94) | 0.01 |
| Oral hypoglycemic agents | 6,732 (33.80) | 9,224 (33.00) | 0.02 | 5,937 (33.62) | 6,007 (33.81) | 0.00 | 6,625.71 (33.27) | 9,400.15 (33.64) | 0.01 |
| SSRI/SNRI | 1,567 (7.90) | 2,139 (7.70) | 0.01 | 1,394 (7.85) | 1,389 (7.82) | 0.00 | 1,535.64 (7.71) | 2,183.79 (7.81) | 0.01 |
| Vitamin K antagonists | 1 (0.00) | 1 (0.00) | 0.00 | 0 (0.00) | 1 (0.01) | 0.01 | 0.73 (0.00) | 1.40 (0.68) | 0.00 |

^*^Assessed by defined daily dose.
Values for categorical variables are presented as n (%), and continuous variables are presented as mean ± standard deviation.
SMD, standardized mean difference; NOAC, Non-vitamin K antagonist oral anticoagulants; ARB, angiotensin receptor blocker; ACEi, angiotensin converting enzyme inhibitor; P-gp, p-glycoprotein; CYP, cytochrome P 450; SSRI, selective serotonin reuptake inhibitors; SRNI, serotonin and norepinephrine reuptake inhibitors

Supplementary Table 3. Sensitivity analysis for risk of the antihypertensive drug-related adverse events

|  | Telmisartan group | | | Olmesartan group | | | Hazard ratio  (95% CI) |
| --- | --- | --- | --- | --- | --- | --- | --- |
|  | PYs | No. of events | IR/1000 PYs | PYs | No. of events | IR/1000 PYs |  |
| Angioedema | 29,556.60 | 21 | 0.70 | 22,106.65 | 13 | 0.60 | 1.17 (0.59–2.32) |
| Hyperkalemia | 29,050.65 | 710 | 24.43 | 21,705.28 | 526 | 24.25 | 0.99 (0.88–1.10) |
| Hypotension | 29,312.43 | 331 | 11.29 | 21,911.24 | 262 | 11.97 | 0.93 (0.79–1.09) |
| Syncope | 28,814.91 | 693 | 24.04 | 21,603.96 | 500 | 23.12 | 1.01 (0.90–1.14) |

PYs, person years; IRs, incidence rates; CI, Confidence Interval

Supplementary Table 4. Sensitivity analysis for negative controls

|  | Telmisartan group | | | Olmesartan group | | | Hazard ratio  (95% CI) |
| --- | --- | --- | --- | --- | --- | --- | --- |
|  | PYs | No. of events | IR/1000 PYs | PYs | No. of events | IR/1000 PYs |  |
| Non-infectious pneumonitis | 29,471.35 | 136 | 4.63 | 22,018.62 | 104 | 4.72 | 0.96 (0.74–1.24) |
| Cancer | 27,962.12 | 1,859 | 66.50 | 20,896.93 | 1,289 | 61.69 | 1.05 (0.97–1.12) |
| Urinary tract infections | 27,869.42 | 1,629 | 58.44 | 20,860.56 | 1,221 | 58.53 | 0.98 (0.91–1.06) |

PYs, person years; IRs, incidence rates; CI, Confidence Interval

Supplementary Table 5. Subgroup analysis for ischemic stroke

|  | Telmisartan group | | | Olmesartan group | | | Hazard ratio |
| --- | --- | --- | --- | --- | --- | --- | --- |
|  | PYs | No. of events | IR/1000 PYs | PYs | No. of events | IR/1000 PYs | (95% CI) |
| **Age groups** |  |  |  |  |  |  |  |
| 65 years | 7,470.13 | 30 | 3.96 | 5,593.49 | 24 | 4.21 | 0.93 (0.54–1.60) |
| 65-74 years | 11,684.90 | 84 | 7.18 | 8,678.24 | 57 | 6.55 | 1.08 (0.77–1.51) |
| 75 years | 10,275.93 | 138 | 13.43 | 7,741.33 | 116 | 15.03 | 0.88 (0.69–1.13) |
| **Sex** |  |  |  |  |  |  |  |
| Male | 17,413.89 | 137 | 7.89 | 13,176.97 | 94 | 7.13 | 1.09 (0.84–1.41) |
| Female | 12,017.07 | 114 | 9.50 | 8,836.09 | 103 | 11.62 | 0.81 (0.62–1.06) |
| **Comorbidities** | |  |  |  |  |  |  |
| Heart failure |  |  |  |  |  |  |  |
| No | 19,670.93 | 148 | 7.53 | 14,550.76 | 123 | 8.47 | 0.88 (0.69–1.12) |
| Yes | 9,760.03 | 103 | 10.59 | 7,462.30 | 73 | 9.84 | 1.05 (0.78–1.42) |
| Renal disease other than end stage renal disease | | |  |  |  |  |  |
| No | 26,727.93 | 215 | 8.04 | 20,034.59 | 170 | 8.46 | 0.94 (0.77–1.15) |
| Yes | 2,703.02 | 37 | 13.53 | 1,978.47 | 27 | 13.71 | 0.97 (0.59–1.60) |
| **Clinical risk scores** |  |  |  |  |  |  |  |
| CHA_2_DS_2_-VASc score |  |  |  |  |  |  |  |
| $<$2 for male ($<$3 for female) | 2,331.49 | 7 | 2.83 | 1,672.26 | 8 | 5.07 | 0.55 (0.20–1.53) |
| $\geq$2 for male ($\geq$3 for female) | 27,099.47 | 245 | 9.04 | 20,340.80 | 188 | 9.25 | 0.96 (0.80–1.16) |
| HAS-BLED score |  |  |  |  |  |  |  |
| $<$3 | 15,988.00 | 105 | 6.56 | 11,951.72 | 95 | 7.98 | 0.81 (0.61–1.07) |
| $\geq$3 | 13,442.95 | 147 | 10.90 | 10,061.34 | 101 | 10.07 | 1.07 (0.83–1.38) |
| **Comedications** | | |  |  |  |  |  |
| P2Y12 inhibitors and other antiplatelet drugs | | |  |  |  |  |  |
| No | 8,597.34 | 69 | 8.01 | 6,600.28 | 49 | 7.42 | 1.05 (0.73–1.51) |
| Yes | 20,833.62 | 183 | 8.76 | 15,412.78 | 148 | 9.58 | 0.91 (0.73–1.12) |
| Number of classes of antihypertensive drugs | | |  |  |  |  |  |
| 1 | 1,322.77 | 5 | 3.96 | 1,217.15 | 10 | 8.37 | 0.47 (0.16–1.35) |
| $\geq$2 | 28,108.18 | 246 | 8.76 | 20,795.91 | 187 | 8.97 | 0.96 (0.80–1.16) |
| **Medication adherence^*^** | |  |  |  |  |  |  |
| $<$0.8 | 156.83 | 7 | 46.66 | 114.40 | 11 | 96.25 | 0.49 (0.19–1.24) |
| $\geq$0.8 | 29,274.12 | 244 | 8.34 | 21,898.66 | 186 | 8.48 | 0.97 (0.80–1.17) |

^*^Calculated by dividing number of days of drug in period covered by number of days in period
PYs, person years; IRs, incidence rates; CI, Confidence Interval

Supplementary Table 6. Subgroup analysis for primary outcome by type of direct oral anticoagulant use

|  | Telmisartan group | | | Olmesartan group | | | Hazard ratio  (95% CI) | P for interaction |
| --- | --- | --- | --- | --- | --- | --- | --- | --- |
|  | PYs | No. of events | IR/1000 PYs | PYs | No. of events | IR/1000 PYs |  |  |
| **Composite of intracranial hemorrhage or gastrointestinal bleeding** |  |  |  |  |  |  |  | 0.63 |
| Dabigatran | 2,102.02 | 14.73 | 7.01 | 1,414.29 | 4.13 | 2.92 | 2.38 (0.80- 7.09) |  |
| Rivaroxaban | 8,403.54 | 87.62 | 10.43 | 6,368.75 | 46.15 | 7.25 | 1.41 (0.99 – 2.02) |  |
| Apixaban | 7,916.62 | 71.29 | 9.00 | 5,371.53 | 41.32 | 7.69 | 1.18 (0.80 – 1.73) |  |
| Edoxaban | 11,007.89 | 95.09 | 8.64 | 8,863.41 | 55.79 | 6.29 | 1.36 (0.98 – 1.90) |  |

PYs, person years; IRs, incidence rates; CI, Confidence Interval

Supplementary Figure 1. Flow diagram

DOACs, direct oral anticoagulants; AF, atrial fibrillation; OACs, oral anticoagulants; ARBs, angiotensin II receptor blockers

Supplementary Figure 2. Propensity score distributions in telmisartan and olmesartan groups

The propensity score was used to balance the distribution of baseline covariates between the two groups.
